# Supplementary material for: Mechanical Stretch Disrupts Calcium Dynamics and Redistributes Piezo1 in Human Astrocytes
Source: Ann Biomed Eng. 2026 Feb 5;54(6):1652–64. doi: 10.1007/s10439-026-03995-0 (PMC13186902; doi:10.1007/s10439-026-03995-0)
Supplement: Supplementary file 1 — Supplementary file1 (DOCX 1249 kb) [file 10439_2026_3995_MOESM1_ESM.docx]

**Supplementary data**


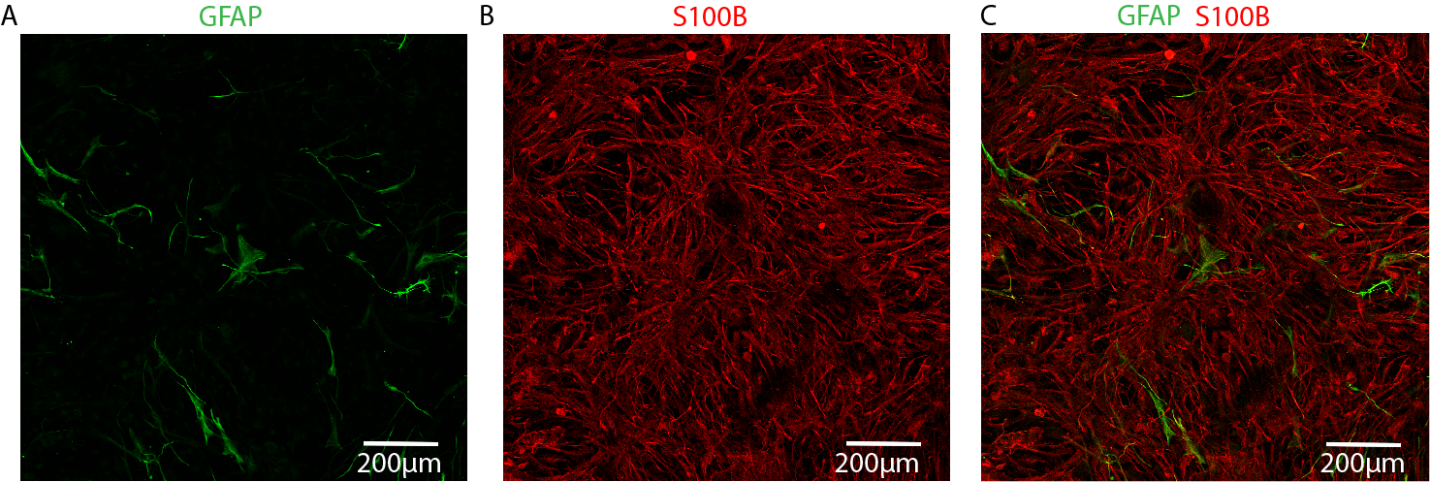


**Figure S1:** Immunostaining for (A) GFAP (B) S100B and (C) Merged in 21 day old astrocytes. Images are representative images of 3 wells.

**
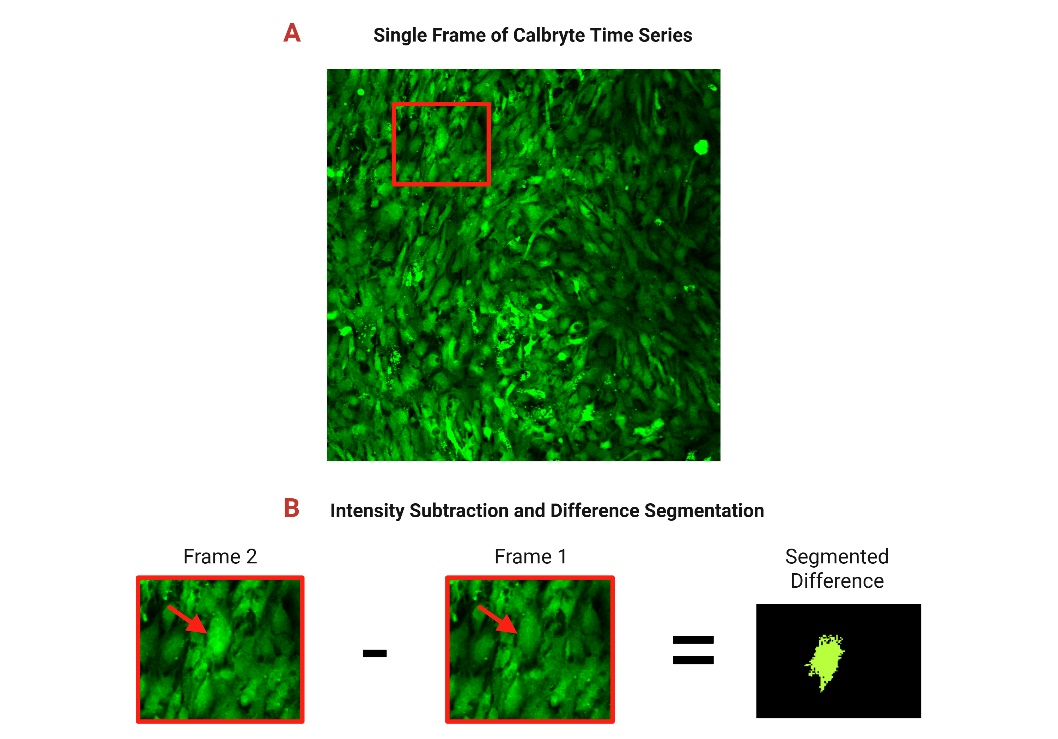
**

**Figure S2:** Workflow of asynchronous activity index (AAI) segmentation. (**A**) Single frame from a Calbryte time series of a sham culture. Red box indicates the region selected for visualization. (B**)** Intensity subtraction and difference segmentation. The cropped region from Panel A (left) and the corresponding region from the previous frame (middle) are shown with red arrows indicating areas of fluorescence change. Regions with larger intensity differences are segmented into discrete objects (right) for further analysis.


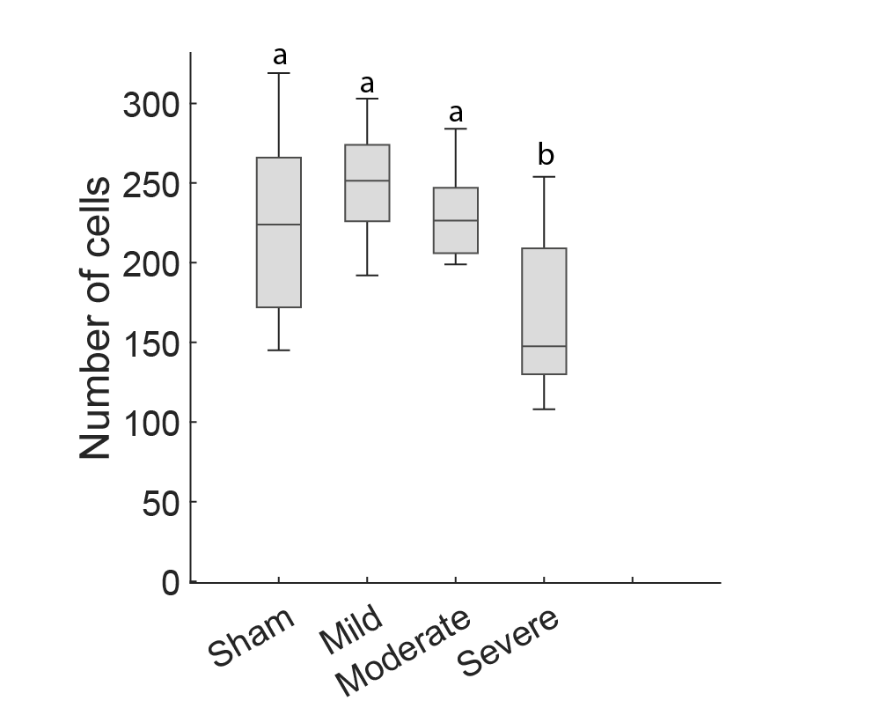


**Fig S3**. Number of cells 24 hours after injury. Box plots display the median and interquartile range (25th–75th percentiles).Whiskers represent the range of values within 1.5 times the interquartile range (N=6 wells). Groups that do not share a letter are significantly different (Kruskal-Wallis test , p< 0.05).


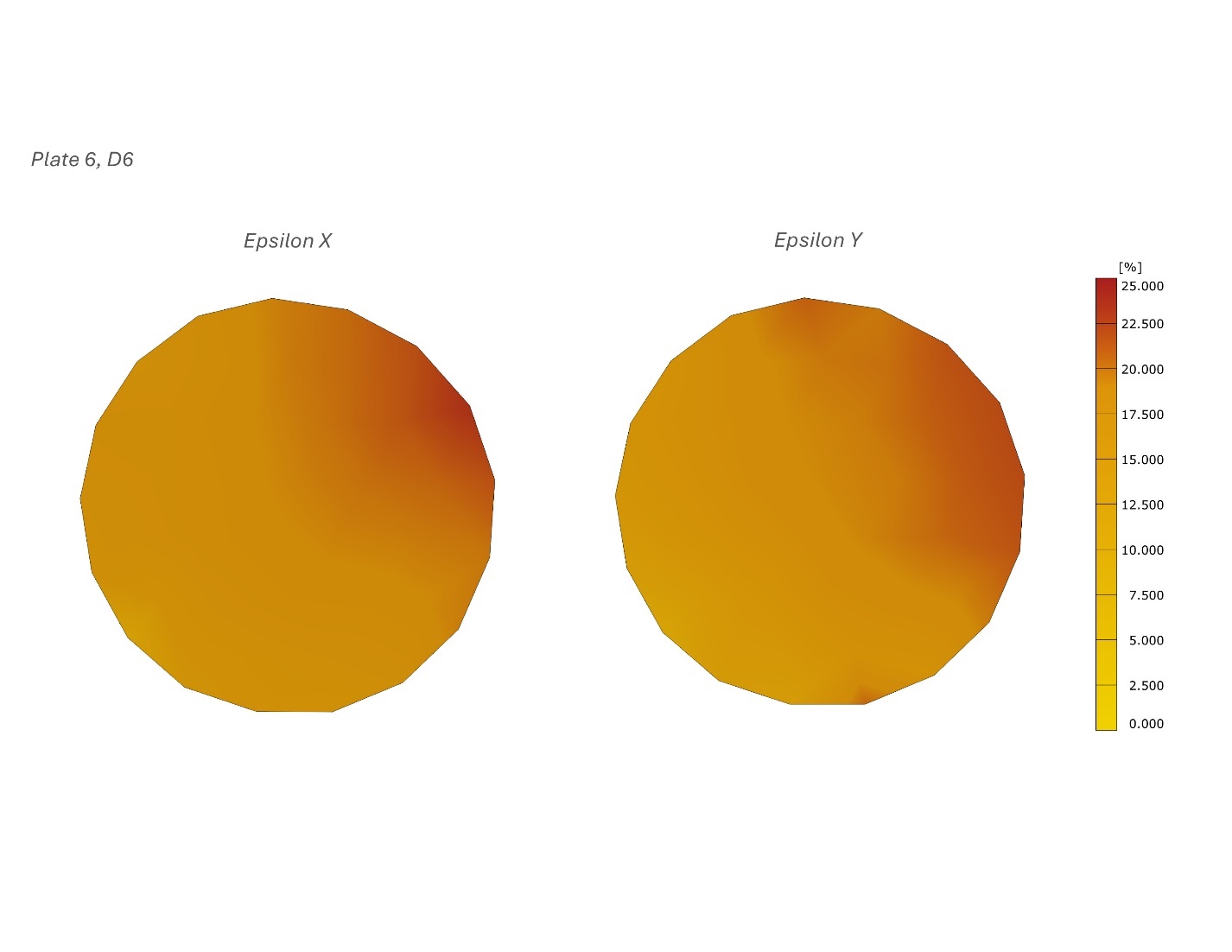


**Fig S4.** Peak strain distributions in X and Y directions for a 1.5 mm deep indentation. (Representative image of 21 wells).


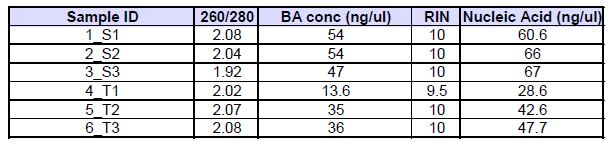


**Table S1.** RNA-quality information for all sequenced samples (S1, S2, S3 are sham and T1, T2, T3 are trauma samples).
